# Teaching medicine through film: Wiseman's medical trilogy revisited

**DOI:** 10.1186/s12909-019-1819-0

**Published:** 2019-10-22

**Authors:** Eelco F. M. Wijdicks

**Affiliations:** Department of Neurology, Mayo Clinic, 200 First Street SW, Rochester, MN 55905 USA

**Keywords:** History of Medicine, Medical ethics, Access to healthcare, End-of-life decision-making, Informed consent

## Abstract

**Background:**

Between the late 1960s and early 1980s, Frederick Wiseman filmed hundreds of hours in an emergency department, intensive care unit and asylum. These films recorded events as they happened without rehearsal and narration.

**Main body:**

Cinema and Medicine meet each other in feature fiction film and in documentary format. Showing films in hospitals is revealing for both the unexpected audience but also the medical establishment. This paper revisits Wiseman’s edited but explicit films and their revelation of the complexity of care in this era in the United States. Although they offer a narrow view of medical institutions and the issue of informed consent later became problematic, the films provide an intriguing glimpse of US healthcare and decision making. These films are largely unknown but would be an invaluable resource in a masterclass on medical ethics in urgent care and end-of-life decisions.

**Conclusions:**

Despite their flaws, Wisemans’ medical films have a significant educational value. Each documentary can be used in a masterclass on medical ethics. The films provide ample opportunities to discuss core issues in healthcare, professional interactions, and decision making in critically ill patients.

## Background

Fictional film directors have used the practice of medicine as a simulacrum. Cinema may, however, search for medical authenticity in documentary filmmaking, and when it does, the healthcare woes are alluring. The disappointing economics of healthcare provisions in the United States were mostly on display in Michael Moore’s *SICKO* [1]*,* released in 2007. *The Waiting Room* [2]*,* a more recent (2012) documentary feature, suggests the toxic situation of overcrowding in the emergency room is the new norm. Where do we go to see a different approach? Where do we find the staples of medical documentaries? Several decades have passed since law professor-turned-filmmaker Frederick Wiseman featured his chosen medical institutions in landmark documentaries: *Titicut Follies* [3], *Hospital* [4]*,* and *Near Death* [5]. In this article we ask the question: Have these three works remained relevant to medical and lay audiences despite the passage of time?

## The documentaries in detail

Despite their genre classification as *documentaries*, Wiseman‘s films are less than entirely true-to-life—he selected a fraction of the hours of material shot over a typical 12-week period and edited it to shape his narrative. He understands too well that this selection supports his own preconceptions, and we can expect confirmation bias to slip in. Physicians had no control over his editing, but he used physicians to present and clarify the medical issues. Deliberately and more cinematically, there is no narration, and no explanation. His attitude towards the healthcare options in the United States—when poor, impaired and destitute —was profoundly negative.

### Titicut follies

*Titicut Follies* (1967) was filmed in the Bridgewater State Hospital, an institution for the criminally insane. Massachusetts Superior Court Judge Harry Kalus ordered the film recalled from distribution with all copies destroyed a year after it was shown. In 1969, the Massachusetts Supreme Court allowed it to be shown only to doctors, lawyers, judges, healthcare professionals, social workers, and students in these and related fields. The censorship, which was lifted in 1991, was ordered on the judicial grounds that the film violated the patients’ rights to privacy; certainly, this seems ironic in an environment in which patients had virtually no rights.

The Court was particularly offended by the portrayal of nudity and the brutal display of forced feeding. Viewing this famous forced-feeding scene again, one must agree that the roughly placed nasal tube is, indeed, “forced,” even by the standards of the time (and certainly by ours). Before the procedure, the patient refuses again and again to drink the sustenance voluntarily but then lies down, seemingly in anticipation of this intervention. The shocking detachment and nonchalance of the attending psychiatrist strike the wrong note in any medically trained viewer, but certainly the psychiatrist has few other options for treating an apathetic, critically starved patient.

*Titicut Follies* show a difficult practice— forensic psychiatry. Another stand-out opening scene, which does not drive the narrative, is an interview with a young paedophile about his sexual fantasies and escapades. Later, a forum of psychiatrists must decide how to help and better medicate a patient with an exaggerated sense of self–confidence who wants to leave but has no insight regarding his major mental disorder. The audience by now must assume that institutions—even those in “the land of the free”— are rigid bureaucracies. Many patients seem either under- or overmedicated, which may have played a role in their mannerisms. A brief scene shows a group of severely affected patients with (likely) congenital neurologic deficits. Few psychiatrists were on staff to provide oversight, and the institution employed guards rather than specialized psychiatric nurses.

What did the critics think? The Museum of Modern Art (MoMA) retrospective of Wiseman’s work is a good guide, and the bundled essays coalesce into a number of observations [6]. Critics have decided the documentaries show the scandalous inadequacy of the medical institution, a place like “the underworld”. Many are angered by the displayed insolence and indifference in *Titicut Follies*. Others are outraged over treatment of inmates and feel it supports the anti-psychiatry movement, which posits that people who were just responding to their harsh society were diagnosed as mentally ill and committed for the convenience. The trope of the asylum is a mirror of the world with psychiatrists as the oppressors.

The medical profession should not tiptoe into these arguments. The State Hospital closed soon thereafter. However, it would be presumptuous to claim the film was responsible.

### Hospital

*Hospital* (1970) is about Metropolitan Hospital Center in East Harlem, one of the poorest neighbourhoods with the highest jobless rate in New York City. The film focuses heavily on bureaucracy and lack of communication. Obtaining medical information in the emergency department is difficult, fragmented, ambiguous, and inherently unreliable. *Hospital* shows a dramatic crowding of an Emergency Department, and now many decades later, the situation is not much better, probably worse. Access to healthcare remains a major point of contention in the United States.

The film is a collage of brief doctor-patient encounters. Wiseman also shows a brain anatomy lecture and another autopsy, which are odd choices for a film focused primarily on the hustle and bustle of an emergency department, but Wiseman may have thought it useful to show because many of us may eventually get an autopsy.

The film shows the beauty of a physical examination, and many seasoned physicians seeing the film will lament its near disappearance. It shows physicians going to great lengths to examine a patient and think about the case rather than simply ordering a battery of tests. *Hospital* portrays a resilient - but obviously stretched to the limit - staff working hard to treat alcoholics, drug addicts, prostitutes, intoxicated college kids, and lost children. Many healthcare workers go the extra distance to take care of insurance coverage or to find better places for patients after discharge.

### Near death

*Near Death* (1989) takes us to the medical pulmonary intensive care unit (ICU) in Beth Israel Boston Hospital. The title is a sobriquet for futile intensive care of the terminally ill. It reveals the painful routine of taking care of patients who cannot survive. The family conferences are recognizable to anyone working in the intensive care unit: families confronted with end-stage everything. For hospital-based physicians and intensivists, it is an everyday reality. The patient’s comfort is our goal.

*Near Death* is also the definitive rebuttal to a commonly heard claim that doctors rush end-of-life decisions by showing endless discussions with team members, considering the alternatives, and looking at the grim big picture. These deliberations take place “offstage” and are therefore usually unknown to families of patients, who typically only hear the final recommendations.

What is strikingly evident in the documentary is that decision-making is a lengthy process requiring enormous amounts of patience and reiteration of the medical situation. One healthcare worker says, “There are not very many patients who you (sic) actually saved their lives and made a difference,” referring to the terminally ill patients who come to the ICU. At the end of the 6-h film, there is a surprisingly positive intra-title message stating that *most* patients admitted to intensive care (without a pre-existing terminal illness) actually survive the ICU — encouraging after so much tragedy.

### Challenges and teachable moments

Wiseman’s trilogy of unsparing medical documentaries brings us face-to-face with intensivists, emergency physicians, and psychiatrists at work on life’s major medical challenges. Wiseman’s decision to film in needy places with needy patients is a deliberate editorial choice. The camera caught real hurt with little that could be allayed. It suggests American Medicine moving towards a large-scale bureaucracy and the possibility that sick poor people have no chance.

Wiseman never returned to these institutions, and he likely would not find another receptive medical institution that would allow him to roll the camera all day. Informed consent is different story these days— relying simply on First Amendment protection would not cut it. This is very sensitive material and certainly will leave an audience unsettled, especially because these institutions are in the United States and not an underdeveloped country. Wiseman tells us there is no decent healthcare available for all; there is nothing reassuring.

Wiseman made a huge impression with his chosen method—but on limited audiences. These films have been widely shown in film festivals and museums and on the public broadcasting service but are otherwise unknown to the general public. Using movies as educational tools has been studied [7–9]. A number of pedagogic techniques could be applied in group discussions or a more open Q-and-A forum with experts on the topics. Wiseman’s films might be suitable for a masterclass on medical ethics in urgent care and end-of-life decisions in the intensive care unit. *Titicut Follies* may colour the current discussion in the US to bring back asylums. The documentaries show that urgent care and end-of-life care are lengthy, time-consuming, and require patience. The films provide ample opportunities to discuss physician compassion, communication skills, and management of urgent situations that need containment. Several core points can be discussed that would enhance knowledge and improve caring (Table 1).

**Table 1:** Key Points in Wiseman's Medical Documentaries

Core Educational Points	
*Titicut Follies*	• Role of asylums in forensic psychiatry • Force-feeding controversy • Informed consent • Psychoparmacology in psychiatry • When to revoke privileges
*Hospital*	• Role of ED in healthcare systems • Insurance (and lack thereof) • Bureaucracy in healthcare • Social welfare safety nets • Race and diversity in illness
*Near Death*	• Consensus among healthcare professionals • Shared-decision model • How to communicate that a patient’s condition is irreversible and terminal • How to assist families in accepting a bad outcome • The role for patient autonomy in a serious illness

Another, more generic but no less vital, discussion may follow on how the lay public, including film critics, could wrongly interpret these films and how this can be explained or remedied. Documentary filmmakers may make films on the state of medicine as a pretext for activism and highlighting bad practices. Bias in the care of patients on the bases of gender, race, or socioeconomic status certainly does occur, but it is misleading to suggest that occurrences in one hospital are replicated nationwide. However, these films undoubtedly provide a penetrating insight into the human condition, and each would allow a discussion on healthcare models and how they could better meet the needs of the underserved. The films show the complexity of interactions between healthcare professionals (*Near Death)*, the difficulty in managing chronically ill psychiatric patients with criminal records (*Titicut Follies*), and the demonstration of how medical staff can maintain compassion despite overcrowding and exposure to nearly unsolvable social problems (*The Hospital*). These remain important bioethical topics for discussion.

## Conclusions

Wiseman’s trilogy places significant demands on the viewer. They are selected glimpses, anecdotal glimpses, poignant glimpses, and glimpses of yesterday applicable to today. For most physicians, both the otherworldliness and recognition are —in one word—baffling. For the audience, the movie has no answer, no backstory, and works from the premise is that healthcare is perversely wrong. It is a narrow view of medical institutions, which would shock many if more widely known. In contrast, medical students, fellows and consultants should rediscover these films and use them for educational purposes and debate.

## Data Availability

Not applicable.

## Abbreviations

ICU: Intensive Care Unit
MoMA: Museum of Modern Art (New York)
